# Are Natural or Anthropogenic Factors Influencing Potentially Toxic Elements’ Enrichment in Soils in Proglacial Zones? An Example from Kaffiøyra (Oscar II Land, Spitsbergen)

**DOI:** 10.3390/ijerph192013703

**Published:** 2022-10-21

**Authors:** Joanna Beata Kowalska, Paweł Nicia, Michał Gąsiorek, Paweł Zadrożny, Michał Hubert Węgrzyn, Jarosław Waroszewski

**Affiliations:** 1 Institute of Soil Science, Plant Nutrition and Environmental Protection, Wroclaw University of Environmental and Life Sciences, Grunwaldzka 53, 50-357 Wroclaw, Poland; 2 Department of Soil Science and Agrophysics, University of Agriculture in Krakow, Al. Mickiewicza 21, 31-120 Krakow, Poland; 3 Department of Polar Research and Documentation, Institute of Botany, Jagiellonian University, Kopernika 27, 31-501 Kraków, Poland

**Keywords:** arctic soils, anthropogenic influence, pollution indices, polluted sea aerosol

## Abstract

Arctic soils may hold potentially toxic elements (PTE); PTE can provide evidence of past or recent pollution. In this study, five soil profiles located on Oscar II Land (Kaffiøyra) were studied to (i) evaluate the ecological status of Kaffiøyra’s soils based on the determination of the possible accumulation of PTE using pollution indices; and (ii) determine the possible origin of PTE enrichment (local factors vs. long-range sources) depending on the distance from the sea. The soils were tested with standard soil science methods. The contamination of five soils was assessed by a wide spectrum of pollution soil indices: Enrichment Factor (EF), Geoaccumulation Index (I_geo_), Potential Ecological Risk (RI), Pollution Load Index (PLI), and Probability of Toxicity (MERMQ). EF values calculated based on Cd, Cr, Cu, Pb and Zn content indicated an anthropogenic origin of the pollution. Values of I_geo_ showed the highest pollution with Cd, while CSI and MERMQ values indicated the highest Cd and Pb levels, but only in the soils located closest to the coast. RI values suggested that soils were under a strong or very strong potential ecological risk, whereas PLI confirmed the high probability of soil quality reduction. Enrichment with PTE has been conditioned by both local (natural) and long-distance (anthropogenic) factors. Among the local factors, parent material was highly relevant. The effect of long-distance anthropogenic factors, especially from European, large industrial centres, was manifested by the high content of PTE in soils located closest to the coastlines, delivered by a wet deposition and sea aerosols. The monitoring and assessment of arctic soil quality are useful practices for the verification of the sources of PTE pollution and the development of methods that can contribute to the protection and maintenance of these vulnerable ecosystems.

## 1. Introduction

Over the last few decades, a lot of attention has been devoted to soil ecosystems in the arctic regions [1,2,3,4,5,6,7,8,9,10], mainly due to the current and projected effects of global warming that may contribute to their degradation [11,12]; arctic soils are considered one of the most sensitive to environmental changes [5,7,13,14,15,16,17,18]. Furthermore, soils occupy only a small area of the whole polar geosystem; therefore, their protection and monitoring are considered to be very important [8,18,19,20,21,22]. 

For many decades, arctic soils appeared to be among the least-polluted and least-degraded ecosystems globally [16,23,24], containing only a minor amount of potentially toxic elements (PTE). Previous work has demonstrated that at least some PTE may be stored in permafrost and released when temperatures increase, potentially contaminating the soil and water of arctic areas [25]. The presence of PTE can be related to lithology, since rocks may contain an elevated content of some elements considered to be toxic [25]. 

Proglacial areas, not stabilised with vegetation, consist of an extremely vulnerable environment exposed to anthropogenic impacts of various origins that contribute to the accumulation of contaminants often enriched with PTE [1,26,27]. The increased content of PTE is often related to the long-range transport of pollutants from industrialised European areas [28,29] as well as local activities including, for example, mining [30]. The accumulation of PTE-enriched pollution, therefore, decreases the ecological status of polar soils and causes significant reduction in their quality [15]. Therefore, studies relating to arctic soil quality evaluation are necessary prerequisites to the monitoring and protection of these areas. 

In recent years, the most suitable way to evaluate soil contamination with PTE has been via pollution indices [31,32,33,34,35,36,37]. The biggest advantages of pollution indices are the ability to evaluate the potential sources of PTE and to determine the ecological risk [33,34]. Together with pollution index calculations, the geochemical background is used, which is defined as the natural values of PTE, for example, in parent material or rocks that have not been influenced by anthropogenic activities. The geochemical background allows for the potential differentiation of the natural origin of PTE content from its accumulation through anthropogenic activities.

**Figure 1:**
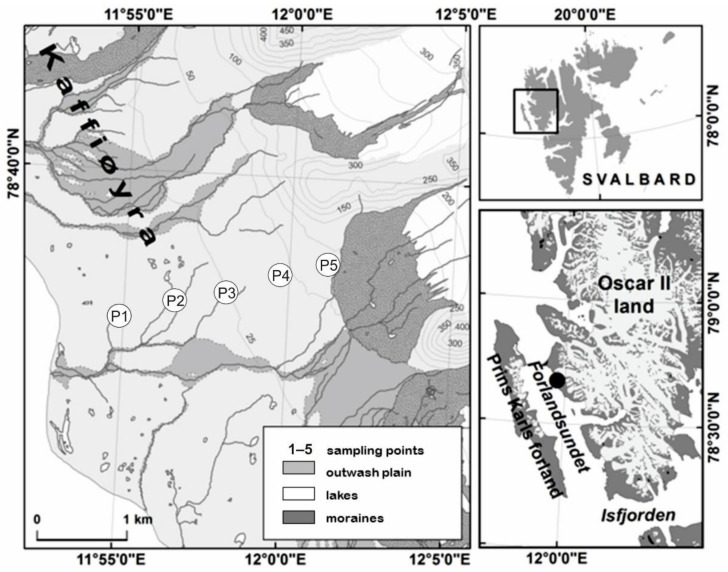
Location of studied soil profiles P1–P5.

The Kaffiøyra plain, located within the Oskar II Land in Svalbard (Figure 1), is an expanding proglacial zone situated at a distance from some pollution sources. The main aims of our study were: (i) to evaluate the ecological status of the Kaffiøyra region’s soils based on the determination of the possible accumulation of PTE using the pollution indices; and (ii) to determine the possible origin of PTE enrichment (local and natural factors vs. long-range sources) depending on the distance from the sea.

## 2. Materials and Methods

### 2.1. Study Area

Field studies were carried out on the Kaffiøyra plain (Figure 1) located on the north-west coast of the Spitsbergen, the largest island of the Svalbard archipelago. A transect of the soil profiles is located between the Irenebreen foreland and the shoreline of Forlandsundet (Figure 1). The Kaffiøyra (78°35’ N, 78°41’ N, 11°50’ E, 12°20’ E) is the coastal terraces plain, being a part of the Oskar II Land. The plain, together with local glaciers, occupies about 310 km^2^, which is only 12% of the total surface of Oskar II Land [6,38]. The Kaffiøyra plain is bounded to the north by the Åavatsmarkbreen sea glacier and to the south by the Dahlbreen sea glacier. The area of the plain is only about 14 km long and 4 km wide, but due to the specific geology, relief, glacier activity and other characteristic features of the natural environment, it creates a very diverse geo-ecosystem [39,40,41].

Two different geological formations comprise the Kaffiøyra terraces. The western part comprises a tectonic Forlandsundet trench of Tertiary origin, while the eastern part is comprised of a crystalline base, folded during the Caledonian orogenesis and elevated to the surface during the Cretaceous–Tertiary transition [42]. The coastal plain is built of Tertiary clay shales, sandstones, conglomerates of silts, dolomites, phyllites and Precambrian and Cambrian marble formations of Hecla Hoek. Their surface is dominated by Quaternary deposits, including coarse-grained deposits such as gravels and sands from marine accumulation, marine and glacial clays, dust deposits and glaciofluvial sediments [43]. Substrates of the weathered Hecla Hoek formation, sandstones and conglomerates of Paleogene origin appear locally [44,45]. The higher sea terraces of abrasive type are covered with boulder-clay material, while the lower sea terraces are built of coastal sediments of the shore zone, gravels, sands and silts [41,42,46]. 

According to other authors [5,21], the study area is dominated by poorly developed, initial soils [20] with thin organic and weakly weathered mineral horizons. Poor soil development is a result of the arctic climatic conditions, which lead to relatively slow rates of chemical weathering and low biological activity [47]. The pedogenesis of arctic soils is often accompanied by cryoturbation or other frost action features [17,48,49]. Haplic Cryosols, developed on the stony and gravelly marine sediments, occur as the main soil type. The loamy parent material provides the substrate for Turbic Cryosols [17]. Moreover, Hyperskeletic Cryosols (Reductaquic) and Turbic Histic Cryosols are characteristic of this region. On the other hand, shallow, sandy or sandy-loam textured Lithic Leptosols can be found [17]. As reported by Plichta and Kuczyńska [16] in the Kaffiøyra area, soils usually hosting gleyic or cambic horizons give rise to the development of Gelic Regosols, Gelic Gleysols or Gelic Cambisols.

Since the end of the Little Ice Age, arctic soils have become exposed due to rapidly retreating glaciers [5,50,51,52,53]. Newly formed proglacial zones with disturbed glaciogenic sediments are unstable [4,54] as permafrost degradation proceeds [40,52,54,55].

The river network of the Kaffiøyra plain, which exists only during the polar summer, creates complex river systems flowing directly from glaciers. Rivers are supplied primarily with waters generated by glacial ablation (57–76.2%), as well as by melting long-term permafrost, a melting snow cover and precipitation [39,56,57,58].

The climate of the Kaffiøyra plain is influenced by the West Spitsbergen Current. January is the coldest month and July is the warmest, with mean monthly air temperatures of −14.2 °C and 4.9 °C, respectively. In the Kaffiøyra region, the mean air humidity is ca. 89%; the mean precipitation during the summer months reaches around 51 mm. 

The Kaffiøyra plain tundra vegetation belongs to the alliance *Luzulion arcticae* and consists mainly of the following species: *Cerastium arcticum*, *Salix polaris*, *Saxifraga cernua*, *S. cespitosa*, *S. oppositifolia*, *Brachythecium glaciale*, *Polytrichastrum alpinum*, *Cetrariella delisei*, *Ochrolechia frigida* and *Cetraria islandica* [59,60,61]. 

### 2.2. Collecting Environmental Data and Soil Samples

The field research was conducted in August 2012. The transect from the Kaffiøyra coastline to Irenebreen glacier (W-NE) includes five soil profiles (P1, P2, P3, P4, P5), at a distance of about 300 m away from each other (Figure 1). Soil profiles have different parent substrates (silt deposits, gravel and sand) and are variously classified according to World Reference Base for Soil Resources [62]. (Cambisols, Regosols and Leptosols with various principal and supplementary qualifiers) (Table 2) [62]. The soil samples were collected in a moist state were placed in polyethene plastic bags for further laboratory procedures. The colour of each separated horizon was determined based on the ‘Revised Standard Soil Color Charts’ [63]. The studied soils were classified according to the World Reference Base for Soil Resources [62].

### 2.3. Laboratory Analysis

Soil samples were air-dried and sieved (sieve mesh diameter 2 mm) before physicochemical analysis. The soil texture was determined using the sieve-hydrometer method, according to the methodology given by Van Reeuwijk [64]. For the determination of pH values, a potentiometric method was applied. Soil pH was measured in a 1:2.5 ratio (*w*/*v*) suspension of H_2_O and 1 M KCl using a standard combination electrode and a pH meter CPI-551 (Elmetron Company, Poland). The content of the total organic carbon (TOC) was determined using the Tiurin method (oxidation of organic matter by a K_2_Cr_2_O7 and H_2_SO_4_ mixture) [65], while the content of total nitrogen (TN) was obtained using a LECO CNS 2000 automatic analyser [66]. 

The content of Cd, Pb, Zn, Cu, Cr and Ni was determined after the wet mineralisation of soil samples in a mixture of concentrated nitric and perchloric acids (2:1 *v*/*v*) [67]. The content of PTE was detected with a Perkin Elmer Optima 7300DV optical emission spectrometer. The following parameters were used during analyses: a plasma gas-flow of 15 dm^3^ min^−1^, external gas flow of 0.2 dm^−3^ min^−1^ and nebulising gas flow of 0.6 dm^3^ min^−1^. A certified multi-element ICP-IV Merck standard solution was used for calibration. The quality of the determination was controlled by the subsequent analysis of GSS-8-certificated reference material (GBW 07408-State Bureau of Metrology, Beijing, China). The detection limits were (mg·kg^−1^): 0.03—Cd, 0.04—Cr, 0.2—Cu, 0.1—Ni, 0.5—Pb and 0.2—Zn.

### 2.4. Pollution Indices

The pollution indices calculated on the basis of absolute PTE content are considered to be a powerful tool for the comprehensive evaluation of soil pollution [33,34,35,36,68,69,70]. To assess the pollution degree of analysed soils, eight pollution indices were calculated: Enrichment Factor (EF) [71], Geoaccumulation Index (I_geo_) [37], Potential Ecological Risk (RI) [32], Pollution Load Index (PLI) [70], Probability of Toxicity (MERMQ) [31] as well as the Contamination Security Index (CSI) [69]. For detailed characteristics, formulae and explanations as well as pollution degrees/classes, see Table 1.

The pollution indices were calculated based on two geochemical backgrounds: (1) local geochemical background—the geochemical composition of the lowermost horizon of each soil; and (2) reference geochemical background—the PTE composition in the upper continental crust (UCC), according to Rudnick and Gao [72]. The Single Pollution Index (PI) values [73] (Table 1) were applied to calculate some of the other pollution indices, such as RI or PLI; therefore, the PI values will not be interpreted in this paper.

For the correct determination of the degree of soil contamination, the selection of the right pollution indices is crucial. In order to avoid uncertainty regarding the assessment of the degree of soil contamination, the authors decided to use a local and a referencel background.

### 2.5. Statistical Analysis

Based on the measured PTE, coefficients of variation (CV) were calculated. In addition, Principal component analysis (PCA) was conducted to define a relationship between the soil properties and PTE content. The reduction in dimensionality was used, which requires a reduction in the number of input variables. The main components were examined based on the projection methods. The normality test was performed to compute the adequacy of the results obtained. The tests and PCA plot were conducted using Statistica 12.5^®^.

The main advantage of PCA is data compression via a reduction from a large number of variables to a small set, yet still containing most of the information. It also allows the data to be easily visualised. A more significant disadvantage of PCA analysis is that the independent variables become less interpretable. A more detailed description of PCA is provided in the paper of Kowalska et al. [34].

## 3. Results

### 3.1. Soil Classification, Morphology and Basic Chemical and Physical Properties

**Figure 2:**
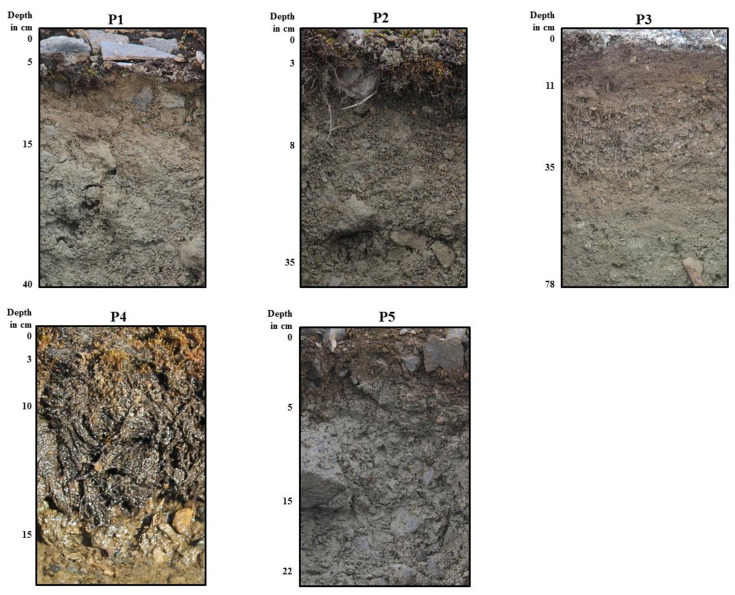
Photographs of analysed soil profiles.

The studied soils were classified into the following reference groups: Cambisols, Regosols and Leptosols (Table 2). Most of the studied soils were relatively shallow (Figure 2), except the soil in profile P3, which was developed on sandy sediments in a floodplain area (Figure 2). All soils revealed a very-firm-to-firm consistency in the lower horizon (Table 2). Weak pedogenic alternations in profiles P1 and P2 allowed for a thin cambic horizon formation. 

**Figure 3:**
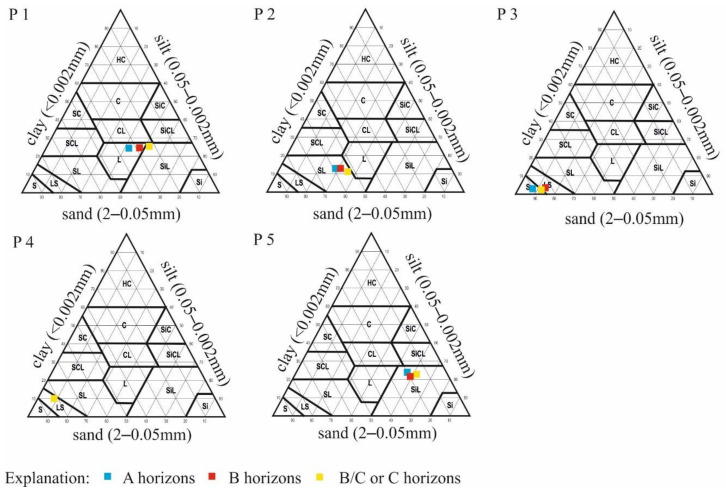
Particle size (mm) distribution (%) of studied soils.

Each soil profile represents a different texture that highlights the variability of parent materials and landforms along the studied transect (Table 2). Profile P1 had a loam texture that overlaid a clay loam, with a quite-high silt content. Profiles P2 and P3 had uniform textures of sandy loam and loamy sand, respectively. The soil in profile P4 developed from moderately decomposed organic material on sandy strata. In profile P5, a clear dominance of silt particles was noted (Table 3, Figure 3).

The pH ranges from 6.3 to 7.9 in H_2_O and from 5.8 to 8.0 in KCl (Table 3). In the investigated soils, pH (both in the H_2_O and KCl solutions) increased with depth (Table 3). In the surface horizons, the lowest pH value was measured in profile P4 (5.8 in KCl solution) while the highest was measured in profile P1 (7.5 in KCl solution; Table 3). All studied soils were characterised by their highest content of TOC and TN in the surface horizons (from 10.2 to 321 g·kg^−1^ and from 0.42 to 2.35 g·kg^−1^ for TOC and TN, respectively). The C:N ratio ranged from 8.1 to 27.2 and was generally higher in the surface horizons compared to the subsurface and C horizons. P2 was characterised by double the C:N ratio of the other analysed soils (Table 3). 

### 3.2. Content of Potentially Toxic Elements

The content of PTE in the studied profiles is presented in Table 4. In the case of Cd, Pb and Zn, the content of PTE was the highest in the surface horizons of all soils, and ranged from 3.5 (P3) to 21.2 (P1) mg∙kg^−1^, from 28.8 (P2) to 65.9 (P1) mg∙kg^−1^, and from 65.2 (P3) to 148 (P4) mg∙kg^−1^, respectively (Table 4). Their content gradually decreased with increasing depth (Table 4). On the contrary, the content of Cr in all soils gradually increased with depth, but the range was very different in each profile; for example, Cr in P5 ranged from 7.51 to 9.58 mg∙kg^−1^, while in P4, it ranged from 28.8 to 57.2 mg∙kg^−1^. Ni showed almost the same content in all horizons of the P1 and P2 profiles, from 22.0 to 22.5 mg∙kg^−1^ and from 10.2 to 11.9 mg∙kg^−1^, respectively. However, in profiles P3 and P4, its concentrations increased with depth. In soil P5, the Cg1 horizon revealed a slight enrichment with Ni, while in the Cg2 horizon, a significant decrease in Ni was observed. The Cu distribution patterns were very diverse. In soils P2 and P5, a slight decrease in Cu down the soils’ profiles was noted, whereas the middle horizon of P1 revealed a minor increase in the Cu amount. In the case of P3, an almost two-fold lower Cu content in the AC horizon was noted, compared to the A and C horizons. In contrast, within P4, a noticeable increase in Cu within the Oe horizon was found (Table 4). Based on the coefficients of variation (CV), the content of Cd, Cr, Ni and Pb were characterised by high variability, while Zn and Cu were characterised by an average variability (Table 4). 

The content of PTE also differed in terms of the distance from the coastline and the glacier. The contents of Cd and Pb were very high in soil P1, which was located closer to the coastline (up to 21.2 and 65.9 mg kg^−1^ in A horizon for Cu and Pb, respectively). A relatively high content of Cu and Zn was measured in soil P5, located near the glacier, and reached the values of 31.2 and 98.6 mg kg^−1^ in the A horizon, respectively. However, the highest content of Cr, Cu, Ni, Pb and Zn was noted in P4 (Table 4), which was enriched in organic matter (Table 3). 

### 3.3. Analysis of the Pollution Indices 

#### 3.3.1. EF

**Figure 4:**
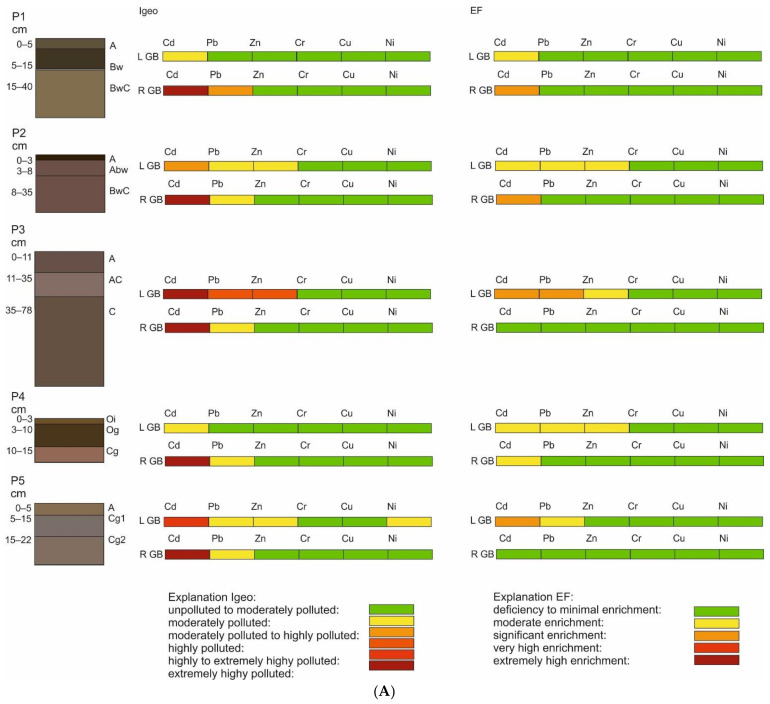

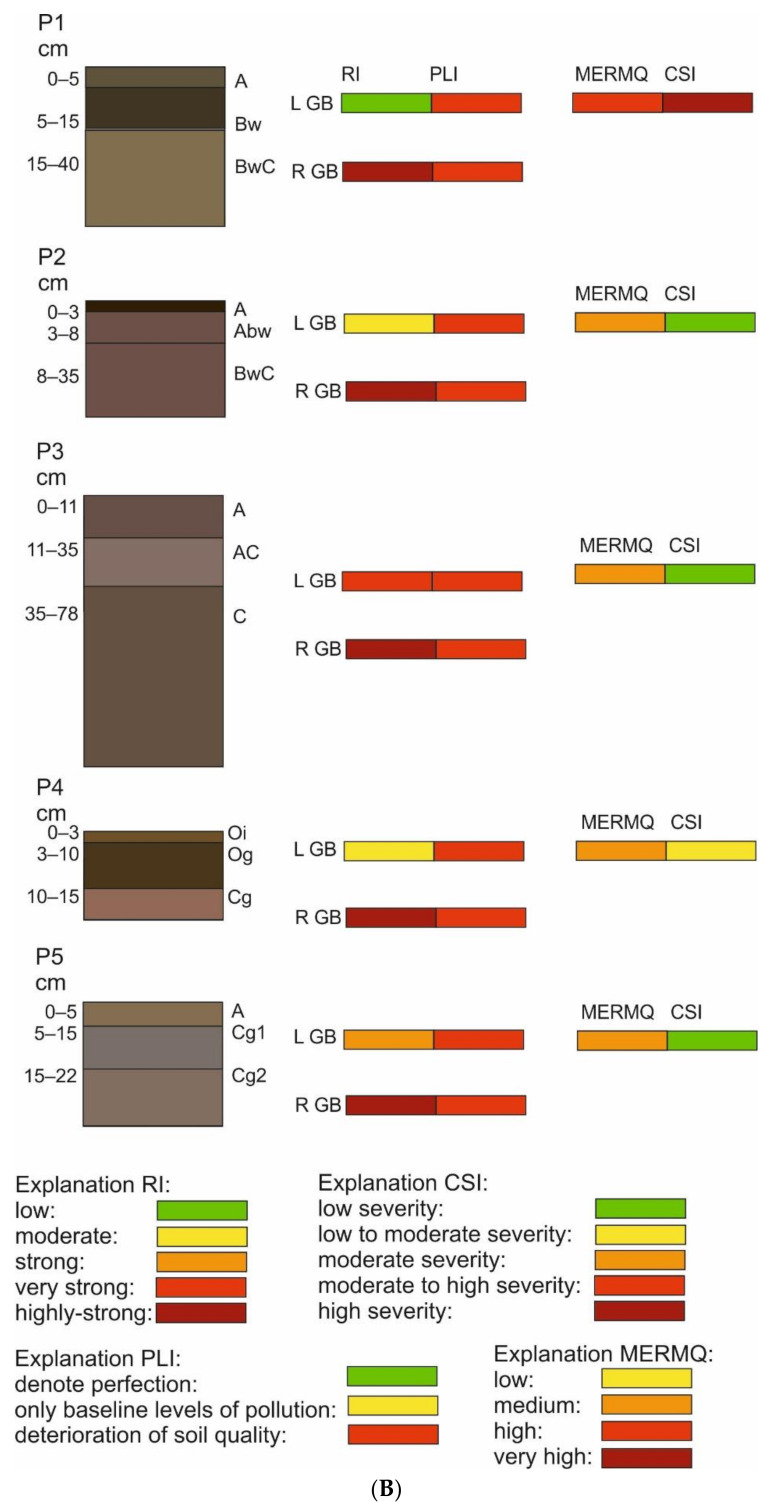
(**A**). The degree of contamination based on pollution indices’ values. Explanation: pollution indices calculated based on: L GB—local geochemical background; R GB—reference geochemical background; I_geo_—Geochemical Index; EF—Enrichment Factor; RI—Potential Ecological Risk. (**B**) The degree of contamination based on pollution indices’ values. Explanation: pollution indices calculated based on: L GB—local geochemical background; R GB—reference geochemical background; RI—Potential Ecological Risk; PLI—Pollution Load Index; MERMQ—The Probability of Toxicity; CSI—Contamination Security Index.

The values of EF were varied and significantly depended on the type of geochemical background used. The PTE enrichment was assessed through the enrichment limits (Table 1). In general, when the local geochemical background was applied, values of EF were relatively higher for most of the studied PTE: Cd, Cr, Ni, Pb and Zn. Minimal enrichment was indicated in the case of Cr, Cu and Ni. Similarly, minimal-to-significant enrichment was stated for Cd, Pb and Zn (Figure 4A). The highest values of EF were recognised in soil P3. When the reference geochemical background was applied, the EF values, which were calculated based on all PTE contents, indicated a deficiency to minimal enrichment, with the exception of Cd, where minimal (P3)-to-significant (P1) degrees of EF were noted (Figure 4A). It should be mentioned that according to EF values, regardless of the applied geochemical background, soil P3 was the most PTE-enriched site (Figure 4A). 

#### 3.3.2. I_geo_

The I_geo_ values calculated for each potentially toxic element were quite similar when using both the local and reference geochemical background (Figure 4A). The I_geo_ values indicated an unpolluted-to-moderate degree of contamination for Cr, Cu, Ni, Pb and Zn (Table 1). In the case of Cd, a moderate-to-extremely-high accumulation was detected using both the local and reference geochemical background (Figure 4A). 

#### 3.3.3. RI 

When the local geochemical background was applied, the studied soils indicated a moderate-to-strong potential ecological risk (Figure 4B), whereas a highly strong potential ecological risk was recognised when the reference geochemical background values were applied (Figure 4B). 

#### 3.3.4. PLI

In the case of the PLI values, the choice of geochemical background had no significant influence on the determination of contamination categories. The PLI values suggested a deterioration of soil quality (Table 1), in both the local and reference geochemical backgrounds. The PLI indicated that soils P1 and P3 had the highest degree of soil quality deterioration (Figure 4B, Table 1). 

#### 3.3.5. MERMQ

The values of MERMQ showed that the risk of soil pollution level ranged from low to high (Figure 4B). The probability of toxicity was very high and ranged from 9 to 49%. MERMQ values showed the highest risk level, as well as the highest probability of toxicity, for soil P1 (Figure 4B).

#### 3.3.6. CSI

Values of the CSI index were quite similar in the studied soil profiles. According to Pejman et al. [69], all analysed soil samples showed a low-to-high severity in toxicity (Figure 4B; Table 1).

### 3.4. PCA Analysis

**Figure 5:**
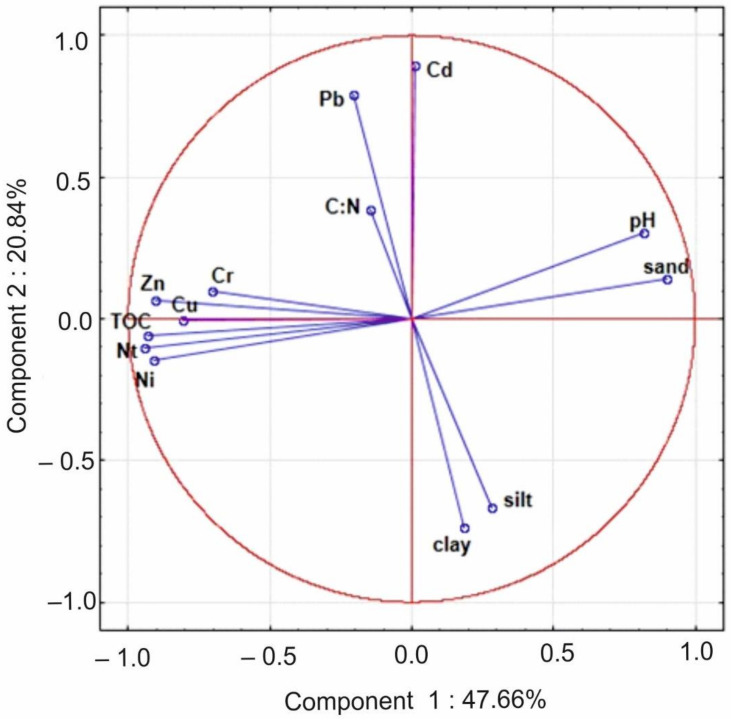
Principal component analysis (PCA) of heavy metal content and other soil variables.

Based on the PCA diagram, the PTE were classified into two groups: Pb and Cd as well as Zn, Cu, Cr and Ni, suggesting two different origins of PTE in the studied soils (Figure 5). The clear relation between Zn, Cu, Cr and Ni with TOC and NT have been stated.

## 4. Discussion

### 4.1. Assessment of PTE Contamination of Kaffiøyra Soils on the Basis of the Analysed Pollution Indices

The arctic region is covered by the Arctic Monitoring and Assessment Programme [75,76], whose purpose is, among others, to monitor and assess the PTE pollution and climate change-related issues. AMAP initiated many years of observations and research that confirmed that arctic soils, similarly to other regions over the world, are also contaminated with PTE [16,77,78,79,80,81,82]. This was the main reason to investigate soil PTE contamination in the studied area, Kaffiøyra. In order to comprehensively assess the PTE contamination, a wide range of pollution indices was used (Table 1). The I_geo_ values (Figure 4A,B) clearly showed that, among the PTE, Cd has the highest degree of contamination in studied soils. Similar I_geo_ results, indicating significant Cd pollution levels in Spitsbergen soils, were reported by Hao [23], Hanaka et al. [83] and Ottesen [84], suggesting the indirect influence of anthropogenic activities; for example, the deposition of airborne pollutants from sources in Europe, North America and Asia on arctic ecosystems (see Section 4.2). 

When the local geochemical background was used in the assessment of the studied soils, enrichment with Cd in each soil profile exceeded the value of 1.0, thus, indicating an anthropogenic source of pollution. Quite high values of EF were determined for Pb and Zn in profile P3, which similarly indicated a moderate contamination resulting from human activity. The anthropogenic input regarding the soils in the arctic areas manifested by PTE enrichment has been described by other authors [23,26,77,85].

The values of CSI and MERMQ confirmed the pollution in profile P1, which was located nearest to the coastline and was, thus, the profile most exposed to a high deposition of sea aerosols, which are often rich in potentially toxic elements. Similar results were described by Gulińska et al. [77], Landing and Paytan [86], Melke [82], Plichta et al. [16], and Węgrzyn et al. [61,87], where the authors unequivocally found a relationship between the distance from the sea and the highest content of PTE, especially in surface soil horizons.

The ecological status of studied soils may be determined using the RI. Based on obtained values, the Kaffiøyra region is under strong or highly strong potential ecological risk. Such a situation may result from a high exposure to external factors, i.e., the modification of chemical compositions by artificial additions (through sea aerosol), as well as internal factors, which include the soil type and its properties [75,76]. In this study, for the areas where RI indicated the highest potential ecological risk, PLI also confirmed the high probability of deterioration of soil quality (Figure 4B). 

The application of different geochemical backgrounds resulted in differences in the pollution indices’ values. In this study, there was a significant difference in terms of applied geochemical background in the pollution classes of I_geo_ and RI values (Figure 4A,B). The application of the local geochemical background gave lower values in those pollution indices. This is related to the fact that the local geochemical background is characterised by spatio-temporal changes conditioned by the difference in PTE in the parent material as well as the physicochemical properties of the soil that, therefore, contribute to a high PTE content in the lowermost horizons [16,33,68,88,89,90]. Moreover, the local geochemical background might be also characterised by increased values due to an occurrence of so-called geochemical anomalies, which are characterised by a high PTE content as result of long and intensive anthropogenic activity in the past [36]. However, a local geochemical anomaly is not supposed to occur within the studied region since Kaffiøyra is an unspoiled area with no past or present intensive industry. Here, the increased content of PTE in the lowermost horizon may be a result of the naturally higher content of PTE in bedrocks. Consequently, higher contents of PTE in the lowermost horizon resulted in lower values of pollution indices. 

On the other hand, when the reference geochemical background was used, the pollution indices’ values (e.g., I_geo_, RI) seemed to be relatively higher. The reference geochemical background was a constant value, characterised by the region and considered a more universal value [33,34,88]. Given the above, there may be a threat of a large discrepancy between the values of indicators. Perhaps the scale of pollution indices, in the case of the reference background application, should be changed so that the pollutant classes would suggest a lower pollution level. However, the application of both geochemical backgrounds gave a complex picture of the possible pollution state of the studied soils and allowed us to estimate the need for further research and monitoring. 

### 4.2. Determination of the Potential Sources of PTE in the Kaffiøyra Region

The studied soils of the recently deglaciated maritime areas of Kaffiøyra are exposed to the impact of PTE accumulation that occur from many different sources [16,43,77,78,79,80,81,82]. In the analysed soils, the content of PTE was higher compared to the topsoil horizons from other arctic regions, for example, from Evseev and Krasovskaya [13], Hanaka [83] and Plichta [16]. The present quality status and enrichment with PTE is an outcome of many agents. First, the local (natural) factors—environmental conditions, specific microclimate, parent materials and soil properties—should be considered. On the other hand, long-distance agents related to anthropogenic activity cannot be ruled out.

PTE enrichment in arctic soils may be linked to glacier retreat [54,91,92,93] and permafrost decline. The glaciers and permafrost can both act as storage, containing a well-preserved record of past atmospheric PTE depositions. During the deglaciation process, PTE previously accumulated under the front of the glacier may be released and contribute to the increasing pools of those elements in newly exposed proglacial zones [4,54]. Similarly, climate warming has contributed to increased leaching and migration of PTE in the permafrost-affected soil cover [25]. Furthermore, the reopened soil surface, previously occupied by glaciers or permafrost, is exposed to further contamination. This phenomenon could be seen in soil P5, located near the forehead of the glacier, where a relatively high content of Cu, Zn and Ni compared to the other investigated soils was noted (Table 4). 

Although the efficiency of organic matter production in tundra areas is quite low, in the arctic region, favourable conditions for the accumulation of organic matter in the topsoil often occur [93,94]. The predominant plant cover mainly consisted of mosses and lichens, which are often able to accumulate significant amounts of air pollutants on the soil surface and limit their movement into the subsoil [61,78]. The impact of plant cover can be clearly observed in the case of Zn, and partially Pb (besides the profile P5; Table 4), and represented the highest values in surface horizons. This is consistent with this widely known relation: the higher the content of organic matter, the greater the soil’s ability to bind pollutants [13,19,68,82,95]. 

A strong positive correlation was found between the analysed PTE and TOC, NT content and C:N ratio (see Figure 5). The highest content of TOC (exceeding 321 g∙kg^−1^ in surface horizons) was noted in the P4 profile, which was classified as Leptic Hemic Leptosol (Gelic). Unambiguously, soil P4 showed the highest contents of most of the studied PTE: Cr, Cu, Ni and Zn (Table 4). The relation between the organic matter and the PTE accumulation in the soil has been also described in other studies related to arctic soils [2,5,19,81,82,95,96,97,98,99].

The distribution of PTE in the soil profiles was varied and reflected not only spatial location and exposure to pollutants but also the chemical composition of the parent material and its weathering [93]. Similarities in the content and distribution of PTE were recognised in the case of soils formed on the same parent material—for instance, in profiles P2 and P3, which were developed on gravels and sand deposits, or in the case of soils P1 and P5, which were formed on silt substrate, where the respective values of Cr, Ni and Pb were very similar (Table 4). On the other hand, the parent material itself can be a potential source of PTE in soils, as observed for P4 (Cr and Ni) and P5 (Cd, Pb, Cu, Ni, Cr) (Table 4). The contents of PTE indicated in the lowest horizons of those soils show much higher values, sometimes several times higher than those in surface horizons, which undeniably suggests the lithological origin of those elements [100]. 

Many authors have reported increased levels of PTE and their accumulation in coastal sediments as the result of long-distance transport [101,102]. Similarly, profile P1 had the highest content of PTE relative to the other studied soils, especially on the surface horizon. Yet, as the studied area is located far away from potential direct sources of PTE, the transfer and accumulation of polluted sea aerosol may have a significant meaning here. Shevchenko et al. [103] noted that concentrations of most PTE in the sea aerosol of the marine boundary layer in the Russian Arctic are almost of the same order as the concentrations in soils from arctic areas. This shows that the contaminants may be transferred even over long distances and may further disturb the soil ecosystems located within the coastal sediments. 

The cross-border transport of pollutants from industrialised areas, especially from Europe and, to a lesser extent, North America, has been noted by other authors [23,26,78,85]. However, in recent years, restrictions connected with dust emissions and heavy industrial production in Eastern Europe have been introduced; for example, the European Pollutant Release and Transfer Register (E-PRTR) [104] for the prevention and control of industrial emissions, Directive 98/70/EC [105], which introduced emission standards for light-duty vehicles, and Directive 2005/55/EC [106], which limited emissions for heavy-duty vehicles; however, in North America, emission limitations are governed (in the U.S.) by the Environmental Protection Agency [107]. Similarly, Canada tracks the number of pollutants (rich in PTE) released into the atmosphere through the Government Agency National Pollutant Release Inventory [108,109]. Even though countries on both continents have significant restrictions concerning metalliferous dust emissions, long-distance industrial pollution may still reach arctic areas, leading to increased PTE in arctic soil ecosystems. 

The process of PTE pollution transfer, especially from large industrial centres over long distances, was described in detail by Mazurek et al. [36], but for terrestrial areas. Considering the various directions of wind blowing from continents to the Svalbard archipelago—for example, W, NE and E, as noted in 2014 [110], and ENE, NW, E and WNW, as noted in 2015 [83]—the transfer and accumulation of PTE-rich pollution from European industrialised regions is highly possible from many directions. This assumption seems to be correct, since Bottenheim et al. [28] reported that aerosol particles have signatures of PTE that can predominantly be traced to Eurasian sources. Furthermore, the high influence of polluted air masses transported from industrialised parts of Europe was found by Steinnes [111]. According to his studies, many PTE, such as Pb, Cd, Zn, As, Sb and Se, have accumulated over long distances in the southernmost Scandinavian area (Norway). This is evidence that the content of PTE in arctic soils may be indirectly conditioned by terrestrial (mainly Eurasian) anthropogenic activity.

Considering the complex system of potential sources of PTE pollution as well as increasing the degradation of natural arctic ecosystems, the continuous monitoring and assessment of soil quality is highly important. It is necessary to maintain the research dealing with PTE content and their distribution within soil profiles prior to seeking a broader perspective and new methods on how to counteract soil degradation. Increasing knowledge on providing a means of assessing threats to soil quality could, therefore, allow for saving these sites and prevent further erosion. Furthermore, keeping track of the extent of anthropogenic interference could offer an opportunity to prevent the destruction of these vulnerable ecosystems.

## 5. Conclusions

The pollution indices used in this study showed PTE enrichment in soils of the area of Kaffiøyra. The obtained values of I_geo_ testified to the highest degree of PTE contamination with Cd. Furthermore, the values of EF confirmed the anthropogenic origin of PTE, but only when the local geochemical background was used. The values of CSI and MERMQ confirmed the highest Cd and Pb pollution in soils located near the coastline (via sea aerosols). Generally, PTE showed highly variable distributions within the profiles, caused by parent material and ongoing soil-forming processes. Based on the obtained results, Kaffiøyra is under a strong or highly strong potential ecological risk. In areas where RI indicated the highest potential ecological risk, PLI also confirmed the high probability of a strong reduction in soil quality.

Enrichment with PTE has been conditioned by both local (natural) and long-distance (anthropogenic) factors. Among the local factors, parent material had the strongest influence. However, the release of the elements during the deglaciation process might also increase Cu, Zn and Ni concentrations in soils located in the vicinity of the retreating glacier. The high content of PTE in soils situated closest to the coastline highlights the significance of long-distance factors on pollution in Kaffiøyra. The studied area was exposed to the deposition of PTE pollution transfer, especially from large industrial centres in Eurasia.

Our study contributes to a better understanding of the state of the environment of the polar regions and an increased public awareness of the need to protect them, especially in light of ongoing climate change.

The continuous monitoring and assessment of arctic soil quality allows for the verification of the source of PTE pollution and development of methods that can contribute to the protection and maintenance of these vulnerable ecosystems.

## Figures and Tables

**Table 1:** Indices of pollution used in this study.

Index	Description and Aim of Use	Formula	Explanations	Limit Values
Enrichment Factor (*EF*)	Assessment of the degree of soil pollution, as well as the possible impact of anthropogenic activities on PTE concentrations in the soil [71].	$EF=\frac{\left[\left(\frac{C}{Fe}\right)\right]sample}{\left[\left(\frac{C}{Fe}\right)\right]background}$	$\text{-}\left[\left(\frac{C}{Fe}\right)\right]sample—\mathrm{content}\;\mathrm{of}\;\mathrm{PTE}\;\mathrm{in}\;\mathrm{analysed}\;\mathrm{sample}\;\mathrm{of}\;\mathrm{soil};\;\text{-}\left[\left(\frac{C}{Fe}\right)\right]background—\mathrm{geochemical}\;\mathrm{background}\;\mathrm{of}\;\mathrm{PTE}\;\mathrm{iron}.$	*EF* classes, according to Sutherland [71]: <2: minimal; 2–5: moderate; 5–20: significant; 20–40: very high; >40 extremely high.
Geoaccumulation Index (*I_geo_*)	The values of *I_geo_* allow the assessment of PTE pollution based on the ratio between the current content of the PTE (in topsoil) and the content of PTE in the bedrock or a specific geochemical background [37].	$I_{geo}=log_{2}\left[\frac{C}{1.5B}\right]$	*C*—PTE current content in topsoil; *B*—content of PTE in the bedrock or geochemical background; 1.5—constant, allowing the analysis of fluctuations of PTE content as a result of natural processes.	*I_geo_* classes introduced by Müller [34]: ≤0: unpolluted; 0–1: unpolluted to moderately polluted; 1–2: moderately polluted; 2–3: moderately to highly polluted; 3–4: highly polluted; 4–5: highly to extremely highly polluted; ≥5 extremely highly polluted.
Potential Ecological Risk (*RI*)	*RI* is an indicator used to assess the degree of environmental risk caused by a concentration of PTE both in water and air, as well as in soil Håkanson [32].	$RI=\sum_{i=1}^{m}Er^{i}\;E_{r}^{i}=T_{r}^{i}xPI$	*E_r_*—single index of ecological risk factor; *m*—number of studied PTE; *T_r_^i^*—the toxicity response coefficient of PTE [32]; *PI*— single pollution index of PTE using reference data according to Rudnick and Gao [72].	Classes of *RI* according to Håkanson [32]: ≤90: low; 90–180: moderate; 180–360: strong; 360–720: very strong; ≥720 highly strong.
Pollution Load Index (*PLI*)	This indicator provides an easy way to prove the deterioration of the soil as a result of the accumulation of PTE [34,70].	$PLI=\sqrt[{n}]{PI_{1}\times PI_{2}\times PI_{3}\times \ldots .PIn}$	n—number of analysed PTE; PI—calculated values of the Single Pollution Index.	<1 denotes perfection; 1 only baseline levels of pollution; >1 deterioration of soil quality.
Single Pollution Index (*PI*)	*PI* is helpful in the assessment of the most dangerous PTE within the studied elements [34]; *PI* is also used in the calculation of, e.g., Potential Ecological Risk (*RI*) [34].	$PI=\frac{C}{B}$	*Ci*—determined PTE content in the layer; *B*– geochemical background according to Rudnick and Gao [72].	Evidence of contamination are values higher than 1.0.
The Probability of Toxicity (*MERMQ*)	*MERMQ* is used as a useful tool to recognize PTE’s harmful impact [34].	$MERMQ=\frac{\sum_{i=1}^{n}\frac{Cn}{ERM}}{n}$	Cn—concentration of each analysed PTE; ERM—values [34] n—number of analysed PTE.	<0.1 low; 0.1–0.5 medium; 0.5–1.5 high; >1.5 very high.
Contamination Security Index (*CSI*)	*CSI* informs about the intensity of concentration of PTE in the soil [69]. In order to calculate the CSI, the effects range low (ERL) and effects range median (ERM) should be used [60]. *CSI* is also helpful to determine the limit of toxicity above which the adverse impact on the soil environment is observed.	$CSI=\overset{n}{\underset{i=1}{\sum }}w$	W—weight of each PTE according to Pejman et al. (2015); C—concentration of PTE; ERL, ERM values [34].	<0.5: uncontaminated; 0.5–1: very low severity; 1–1.5: low severity; 1.5–2: low to moderate severity; 2–2.5: moderate severity; 2.5–3: moderate to high severity; 3–4: high severity; 4–5: very high severity; >5: ultra-high severity.

**Table 2:** A morphological description of studied soils and their systematic position according to the WRB classification [62].

Profile	Horizon	Depth (cm)	Parent Material	Colour * (Moist)	Boundary	Structure	Consistence (Moist)	Skeleton Grains	Moisture	Abundance of Roots	Soil Classification
P1	A	0–5	Silt deposits	2,5Y 4/2	A	VF SB MO	FR	A	M	V	Eutric Skeletic Leptic Cambisol (Gelic)
	Bw	5–15		2,5Y 3/2	C	VF SB ST	FI	A	M	N	
	BwC	15–40		2,5Y 5/3		FI SB MO	VFI	A	M	N	
P2	A	0–3	Gravels and sands	10YR 2/3	A	ME GR MO	FR	A	M	M	Eutric Skeletic Leptic Cambisol (Gelic Humic)
	ABw	3–8		10YR 4/4	C	VF SB MO	FI	A	M	V	
	BwC	8–35		10YR 4/4		VF SB MO	VFI	A	M	N	
P3	A	0–11	Gravels and sands	2,5YR 4/2	A	SG	FR	M	SM	C	Eutric Sketetic Regosol (Gelic Humic)
	AC	11–35		2,5YR 5/2	C	FI GR WE	FI	D	SM	C	
	C	35–78		7,5Y 4/2		ME GR MO	FI	A	M	N	
P4	Oi	0–3	Organic matter and sands	10YR 4/4	A	D3	LO	N	W	M	Dystric Histic Leptosol (Gelic)
	Oe	3–10		10YR 3/3	C	D4	LO	VF	W	F	
	Cg	10–15		2,5Y 5/4		SG	FR	D	W	N	
P5	A	0–5	Silt deposits	10YR 5/3	A	FI SB WE	FR	A	SM	N	Eutric Skeletic Gleyic Regosol (Gelic)
	Cg1	5–15		5Y 5/1	C	FI AB ST	VFI	A	M	N	
	Cg2	15–22		5Y 5/2		FI AB ST	VFI	A	M	N	

Explanations: * according to Oyama and Takehara [63]; Boundary [74]: A- abrupt; C—clear; Structure [74]: (1) Size classes: VF– very fine; ME—medium, (2) Types of structure: SG—single grain; AB—angular blocky; SB—subangular blocky, (3) Classification of structure: WE—weak; MO—moderate; ST—strong; Consistence [74]: FR—friable, FI—firm, VFI—very firm; Skeleton grains: A—abundant; M—many; D—dominant; VF—very few; Moisture [74]: SM—slightly moist; M—moist, W–wet; Abundance of roots [74]: N—none; V—very few; F—few; C—common; M—many.

**Table 3:** Basic chemical and physical soil properties.

Profile	Depth	Horizon	pH		TOC	TN	C:N	Coarse Fragments (%)	Texture *
	(cm)		H_2_O	KCl	g·kg^−1^			>2.0	
P1	0–5	A	7.9	7.5	11.6	0.45	25	50	L
	5–15	Bw	7.7	7.5	7.58	0.36	20	30	L
	15–40	BwC	7.8	7.6	4.51	0.24	18	30	SiL
P2	0–3	A	7.3	6.9	47.2	1.04	45	50	SL
	3–8	ABw	7.8	8.0	19.1	1.14	16	45	SL
	8–35	BwC	7.9	7.9	18.2	1.12	16	40	SL
P3	0–11	A	6.7	6.6	48.2	2.35	20	20	S
	11–35	AC	7.3	7.2	25.6	1.45	17	95	LS
	35–78	C	7.6	7.2	9.58	0.49	19	60	LS
P4	0–3	Oi	6.3	5.8	321	12.7	25	-	-
	3–10	Oe	6.5	5.9	245	12.0	20	-	-
	10–15	Cg	7.2	6.4	88.5	4.54	19	5	LS
P5	0–5	A	7.0	6.7	10.2	0.42	24	90	SiL
	5–15	Cg1	7.2	7.0	6.52	0.24	27	95	SiL
	15–22	Cg2	7.2	7.1	1.14	0.14	8	95	SiL

* Explanations: L—loam; SiL—silty loam; SL—sandy loam, S—sand; LS—loamy sand.

**Table 4:** Total content of PTE.

Profile	Depth (cm)	Horizon	Cd	Cr	Cu	Ni	Pb	Zn
			mg∙kg^−1^					
P1	0–5	A	21.2	31.2	30.4	22.2	65.9	75.5
	5–15	Bw	17.5	32.1	31.2	22.0	64.2	72.3
	15–40	BwC	10.2	32.3	30.2	22.5	64.2	65.4
P2	0–3	A	9.35	10.9	21.0	11.2	28.8	79.4
	3–8	ABw	3.95	11.2	19.3	10.2	13.6	58.6
	8–35	BwC	2.38	12.8	19.7	11.9	11.1	47.5
P3	0–11	A	8.35	12.3	10.2	8.35	29.3	65.2
	11–35	AC	4.64	13.2	5.68	9.35	24.2	41.2
	35–78	C	0.74	14.5	12.2	12.3	5.42	21.1
P4	0–3	Oi	5.90	28.8	39.2	48.8	32.5	148
	3–10	Oe	3.27	47.5	54.5	67.7	32.4	125
	10–15	Cg	2.27	57.2	42.1	64.4	27.3	115
P5	0–5	A	3.50	7.51	31.2	16.5	29.3	98.6
	5–15	Cg1	0.89	8.24	30.2	17.2	25.2	84.3
	15–22	Cg2	0.42	9.58	30.2	10.2	14.2	45.2
CV *			97.5	70.2	47.3	84.1	61.6	44.9

Explanations: * coefficient of variations.

## Data Availability

Not applicable.
